# Survival-weighted quality of life profiles in patients treated for laryngeal and hypopharyngeal cancers

**DOI:** 10.7150/jca.92400

**Published:** 2024-02-04

**Authors:** Yao-Te Tsai, Ku-Hao Fang, Wen-Cheng Chen, Andrea De Vito, Chun-Ta Liao, Chung-Jan Kang, Cheng-Ming Hsu, Ethan I. Huang, Ming-Shao Tsai, Geng-He Chang, Yi-Chan Lee, Chia-Hsuan Lai

**Affiliations:** 1 Department of Otorhinolaryngology-Head and Neck Surgery, Chang Gung Memorial Hospital, Chiayi, Taiwan.; 2 College of Medicine, Chang Gung University, Taoyuan, Taiwan.; 3 Department of Otorhinolaryngology-Head and Neck Surgery, Chang Gung Memorial Hospital, Linkou, Taiwan.; 4 Department of Radiation Oncology, Chang Gung Memorial Hospital, Chiayi, Taiwan.; 5 Ear Nose Throat (ENT) Unit, Department of Surgery, Forlì Hospital Health Local Agency of Romagna, Italy.; 6 Department of Otorhinolaryngology-Head and Neck Surgery, Chang Gung Memorial Hospital, Keelung, Taiwan.

**Keywords:** laryngohypopharyngeal cancer, life expectancy, quality of life, quality-adjusted life expectancy, survival-weighted psychometric scores

## Abstract

**Objectives:** This study assessed functional outcomes and quality of life (QoL) in the long term in individuals treated for laryngohypopharyngeal cancer (LHC) by estimating their life expectancy (LE), survival-weighted psychometric scores (SWPSs), and quality-adjusted LE (QALE).

**Materials and methods:** To estimate survival outcomes, we retrospectively reviewed the data of 1576 patients treated for primary LHC between January 2010 and December 2018 and followed them until death or December 2020. We also prospectively collected QoL and functional data between October 2013 and November 2022 from 232 patients by administering the Taiwanese Chinese versions of the QoL Questionnaire Core 30, Head and Neck 35, and EQ-5D-3L. To estimate LE, we employed linear extrapolation of a logit-transformed curve. We calculated QALE and SWPSs by combining the QoL data with the LE results.

**Results:** We estimated the LE of the patients with LHC to be 7.8 years and their loss of LE to be 15.7 years. The estimated QALE was 7.0 QALYs, with a loss of QALE of 16.5 QALYs. Lifetime impairment durations were estimated for cognitive (4.9 years), physical (4.2 years), emotional (3.4 years), social (3.4 years), and role functions (2.7 years). We estimated the durations of problems related to swallowing, speech, and teeth to be 6.2, 5.6, and 4.8 years, respectively. The patients were expected to be dependent on feeding tubes for 1.2 years.

**Conclusions:** Patients with LHC experience significant reductions in both LE and QALE. SWPSs may constitute a valuable tool for obtaining subjective information regarding how LHC affects multifaceted QoL outcomes.

## Introduction

Hypopharyngeal cancer has one of the most unfavorable prognoses of all head and neck cancers (HNCs)^1^. Laryngeal cancer is the most common malignancy within the upper aerodigestive tract^2^. Although survival outcomes are the main focus of cancer management, patients with laryngohypopharyngeal cancer (LHCs) also often experience functional impairments and reduced quality of life (QoL) because of the effects of their tumors on critical anatomical regions and because of the multidisciplinary treatments they must undergo^2^-^7^. Both ablative surgery and radiotherapy (RT)/chemoradiotherapy (CRT) have substantial effects on the QoL of patients with LHC; patients undergoing pharyngolaryngectomy and flap reconstruction frequently experience difficulties in social interactions and role functioning^6^, ^8^, and those undergoing RT often experience long-term problems such as dry mouth and sticky saliva^2^. Because of these common concerns in patients with LHC, studies on the management of LHC have generally assessed functional outcomes and QoL as primary endpoints^2^, ^4^, ^6^.

Assessments of treatment outcomes for patients with HNC are generally conducted from both subjective and objective perspectives. Patients provide subjective accounts that guide assessments of their social, emotional, psychological, and physical outcomes; moreover, clinicians obtain objective observations of their complications, locoregional control, and survival outcomes^9^. The focus on patient-reported outcomes in the literature has gradually increased^4^. Researchers have highlighted the importance of regularly evaluating the QoL of patients with LHC through the administration of questionnaires during and after treatment, reporting that the results of such evaluations can be used to enhance treatment optimization, rehabilitation planning, and communication between clinicians and patients and to achieve better overall outcomes^4^. In LHC, long-term QoL after treatment is a key consideration when clinicians are choosing between different treatment modalities with comparable survival outcomes^10^; clinicians must remain mindful of the lifelong effects of treatments when they offer guidance regarding treatment selection to patients with LHC. The concept of quality-adjusted life expectancy (QALE), in which both QoL and survival outcomes are considered, has gained considerable traction in oncology research and has begun to be used in developing care plans for patients with cancer^11^, ^12^. Several studies have calculated life expectancy (LE) and QALE to evaluate how the disease affects the QoL of patients with HNC^12^, ^13^. Furthermore, a study applied survival-weighted psychometric scores (SWPSs) to obtain findings that extend beyond the period in which patients were followed up, thus obtaining insights into the effects of leukemia on the multifaceted aspects of QoL in the long term^11^. However, no study has calculated SWPSs for patients with LHC, and findings regarding LE and QALE in these patients are also lacking. Accordingly, the present research determined the practicability of estimating SWPSs and QALE for patients with LHC through the use of both survival functions and average QoL scores at different time points.

## Materials and methods

### Research population

**Figure 1:**
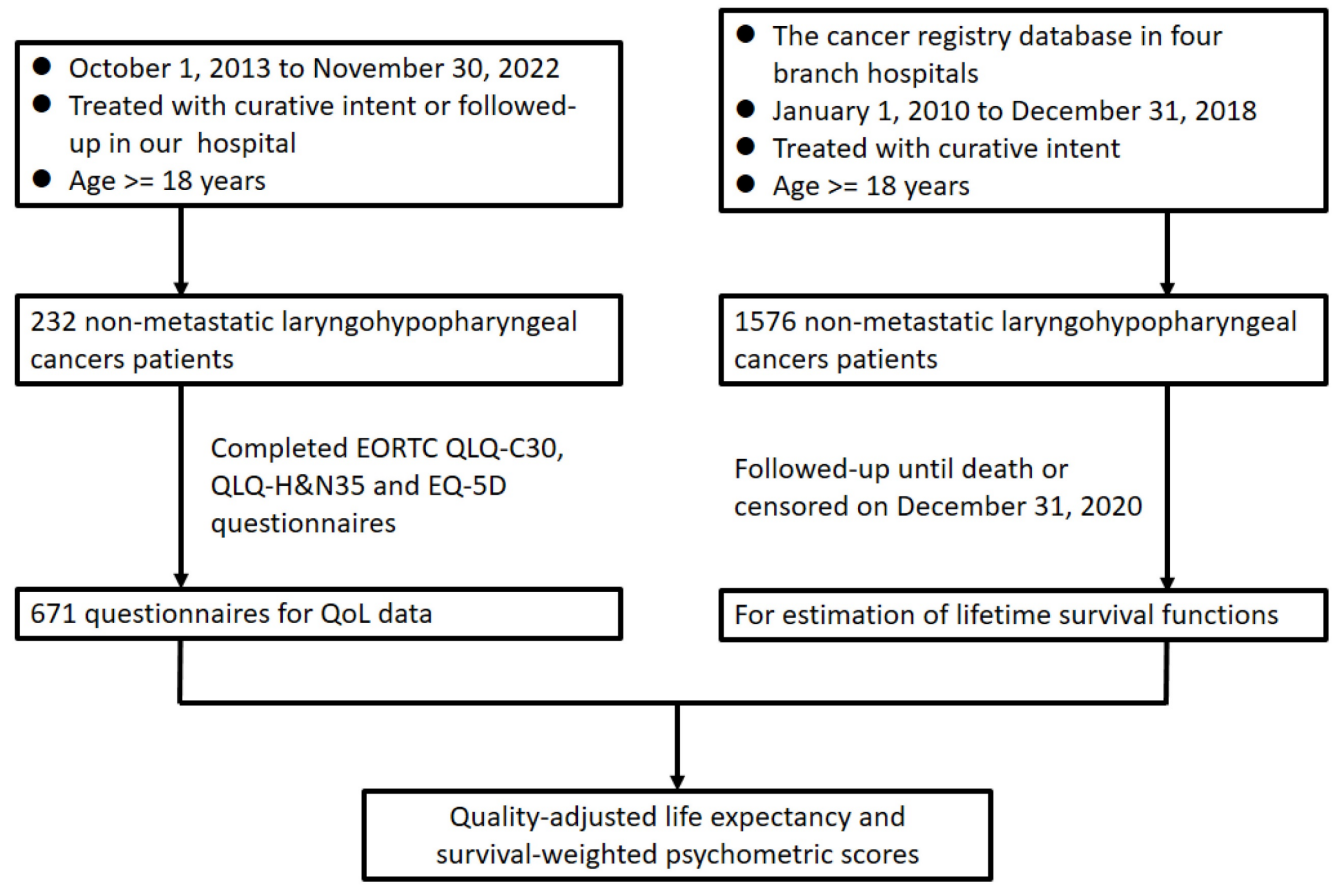
Flowchart of study design. Abbreviations: EORTC, European Organisation for Research and Treatment of Cancer; H&N, head and neck; QLQ, quality of life questionnaire; QoL, quality of life.

A flowchart illustrating the design of this study is presented in Figure 1. To calculate survival outcomes, the data of individuals given a diagnosis of primary LHC between January 2010 and December 2018 were gleaned from our institutional cancer registry database and retrospectively reviewed. Individuals were considered eligible for inclusion in our study if they were 18 to 75 years old, had an LHC diagnosis that had been confirmed histopathologically, had undergone curative treatments at our hospital, and had a score on the Eastern Cooperative Oncology Group Performance Status Scale of 0 to 2. Individuals were considered ineligible for inclusion if they had received palliative care, were reported to have a previous malignancy or to have concurrent cancer at diagnosis, or had incomplete data pertaining to the variables of interest. Accordingly, on the basis of the aforesaid criteria, a total of 1576 patients with LHC were identified for inclusion in our overall survival (OS) estimation. We reviewed and analyzed information in the included patients' medical records, including their sex; age; tumor-node-metastasis (TNM) classification, as determined using the American Joint Committee on Cancer's manual for staging cancer (2010); tumor subsite; forms of treatment; and survival status by the end of November 2022. To obtain QoL data, we prospectively enrolled patients with LHC who met the aforementioned criteria and were regularly followed up at our institution between October 2013 and November 2022. We executed our study in accordance with the principles of the Declaration of Helsinki, with all participants providing written informed consent before participating. Our hospital's institutional review board granted approval for our study protocol.

### Treatment protocol

On the basis of clinical judgment and patient preferences, each patient was subjected to either RT/CRT or ablative surgery with or without subsequent adjuvant therapy. In the primary surgery group, the patients underwent tumor excision using either transoral laryngomicrosurgery with a CO_2_ laser or open (partial or total) pharyngolaryngectomy along with neck dissection and wound reconstruction. Decisions regarding the application of postoperative adjuvant RT or CRT were made through a tumor consensus conference at the institution, and therapy was administered at a frequency of one fraction per day, 5 days per week, with a total of 66 Gy delivered in 33 fractions, if deemed necessary. Patients who underwent definitive or adjuvant CRT received cisplatin-based chemotherapy regimens (100 mg per m^2^ once per week for 3 weeks or 40 mg per m^2^ once per week). All patients underwent pretreatment and posttreatment voice and swallowing rehabilitation. After their final treatment, the patients underwent regular follow-ups. During the first 2 years following their treatment, follow-up appointments were arranged for every 2 months. After the first 2 years, follow-up appointments were arranged every 3 to 6 months. The patients completed questionnaires during their follow-up visits.

### QoL measurement instruments

We evaluated the patients' QoL by using the validated Taiwanese Chinese versions of the European Organisation for Research and Treatment of Cancer (EORTC) QoL Questionnaire Core 30 (EORTC QLQ-C30) and the EORTC QoL Questionnaire Head and Neck 35 (EORTC QLQ-H&N35)^14^, ^15^. EORTC QLQ-C30 comprises a global QoL scale, with three symptom scales, five functioning scales, and six individual items (Table 2). EORTC QLQ-H&N35 is an additional module that complements EORTC QLQ-C30, and it incorporates items designed to assess the QoL of patients with HNC. EORTC QLQ-H&N35 comprises 11 single-item scales and 7 multiple-item symptom scales (Table 2). In accordance with the guidelines of the EORTC scoring manual, all scales are scored within the range of 0 to 100, and these scores are subjected to a linear transformation^16^. On the QoL and functioning scales, scores that are higher indicate that an individual's global QoL and functioning are at higher and healthy levels. Conversely, on the symptom scale, a score that is higher indicates that an individual's problems or symptoms are more severe. Considering that both the EORTC QLQ-C30 and H&N 35 questionnaires included pain symptoms, the pain symptom utilized for the calculation of survival-weighted psychometric scores was derived from the EORTC QLQ-C30 questionnaire. To evaluate QALE, we employed the Taiwanese version of EQ-5D-3L^17^. On this questionnaire, five domains, namely self-care, pain/discomfort, anxiety, activities of daily living, and mobility, are rated as having “no problems,” “some problems,” or “extreme problems.” We converted the results for the aforementioned domains to health-related utility values by applying the time trade-off method^18^. These values ranged from 0 to 1 and represented an individual's level of overall health, with 1 denoting perfect health and 0 indicating death. The questionnaires were administered to patients with the assistance of medical staff.

### Statistical analysis

We present herein the categorical variables as numbers and percentages, and we present the continuous variables as means and standard deviations. Using data from our institution's cancer registration database, we computed the survival times of 1576 patients by measuring the period between when curative treatment began and either December 31, 2020, or the occurrence of death. To estimate OS, Kaplan-Meier curves were generated. In addition, we calculated the survival function for the reference population, obtained using sex and age matching, by employing the Monte Carlo method, with the life-table data of Taiwan's general population used as a basis for calculation^19^. To determine the LE of the patients with LHC, we executed a linear extrapolation of the logit-transformed survival curves between the patients with LHC and the reference population^19^-^21^. Moreover, to derive the average QoL function, we subjected the QoL data of 232 individuals to Kernel smoothing^21^. We graphed the symptoms and functional disabilities at the time that curative treatment was initiated. We subsequently integrated the survival-outcome data and the psychometric scores or utility values for the period spanning from the start of curative treatment to each QoL follow-up data point. Thus, we were able to determine the patients' QALE and SWPSs^9^. We applied the assumption that after the final time at which QoL data were collected, QoL remained constant. We multiplied the utility values or psychometric scores at distinct times by their corresponding survival probabilities. This multiplication resulted in the creation of a survival curve adjusted for QoL. We calculated the area under the curve to determine the patients' SWPSs and QALE^22^. The SWPS of each item was used to estimate the duration of living with a particular psychometric problem after treatment. LE is an estimate of the duration of living, and QALE is an estimated LE adjusted with consideration of an individual having a given level of compromised health at different time points after receiving treatment. For the entire study period, a utility value of 1 was assigned to the reference population. A minimum sample size of 50 was recommended for generating the mean QoL function curve^21^. Factoring in the extrapolation over a 50-year survival period and using 10-year follow-up data, we estimated the QALE, SWPSs, and LE of the patients with LHC. We extrapolated the survival period by employing the iSQoL method (http://sites.stat.sinica.edu/tw/isqol/; validated in previous studies)^11^, ^23^, ^24^. All statistical analyses were conducted in SPSS (version 17.0; SPSS, Chicago, IL, USA), and *p* < 0.05 was considered to indicate significance.

## Results

### Patient characteristics

We present in Table 1 a summary of the characteristics of the patients included in this study. Lifetime survival estimates were derived from survival information obtained for 1576 individuals with LHC. Additionally, a subset of 232 patients was prospectively selected to complete QoL questionnaires. In the overall study population, stage IV LHC was the most common, occurring in 966 patients (61.3%); stage I LHC was the second most common, occurring in 265 patients (16.8%); and stage III LHC was the third most common, occurring in 176 patients (11.2%). For the primary treatment modalities, curative surgery was performed for 523 patients (33.2%). Postoperative adjuvant chemoradiotherapy (CRT) was performed for 175 patients (11.1%), and adjuvant radiotherapy (RT) was performed for 50 patients (3.2%). In addition, 1053 (66.8%) patients received definitive RT (n = 83, 5.3%) or CRT (n = 970, 61.5%) as the primary treatment.

### Survival outcomes regarding LE and QALE

**Figure 2:**
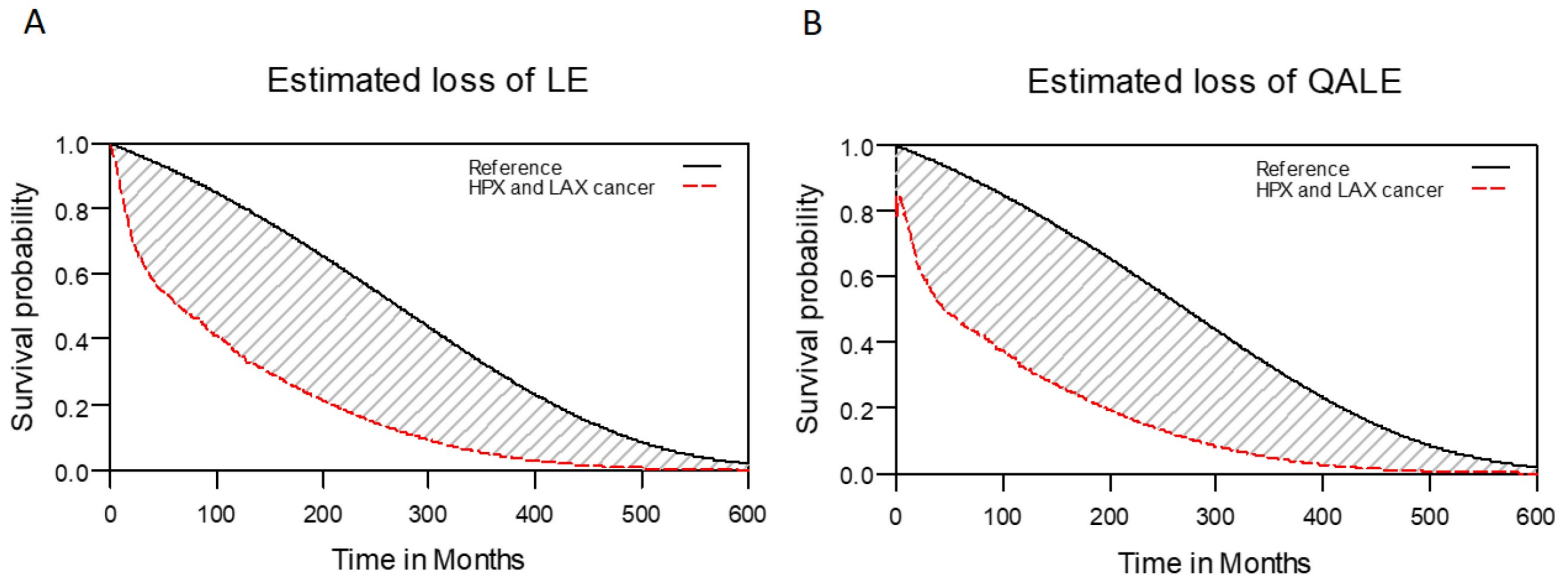
Estimated reduction of LE and QALE in patients with laryngohypopharyngeal cancer. (A) Estimated loss of LE; (B) estimated loss of QALE.

For the 1576 patients given a diagnosis of LHC, we calculated the 5-year OS rate to be 50.4%. The median (range) follow-up duration was 33.9 (0.7 to 131.6) months. For the reference population, we determined the LE and QALE to be 23.5 years and 23.5 quality-adjusted life years (QALYs), respectively. In addition, for the study cohort, the estimated LE was 7.8 years (95% confidence interval [CI]: 6.3 to 11.0 years); the estimated QALE was 7.0 QALYs (95% CI: 5.8 to 10.4 QALYs). These findings indicate that the study cohort had an LE and QALE that were respectively 15.7 years and 16.5 QALYs shorter than those of the reference population (Figure 2).

### Symptoms and impaired function

**Figure 3:**
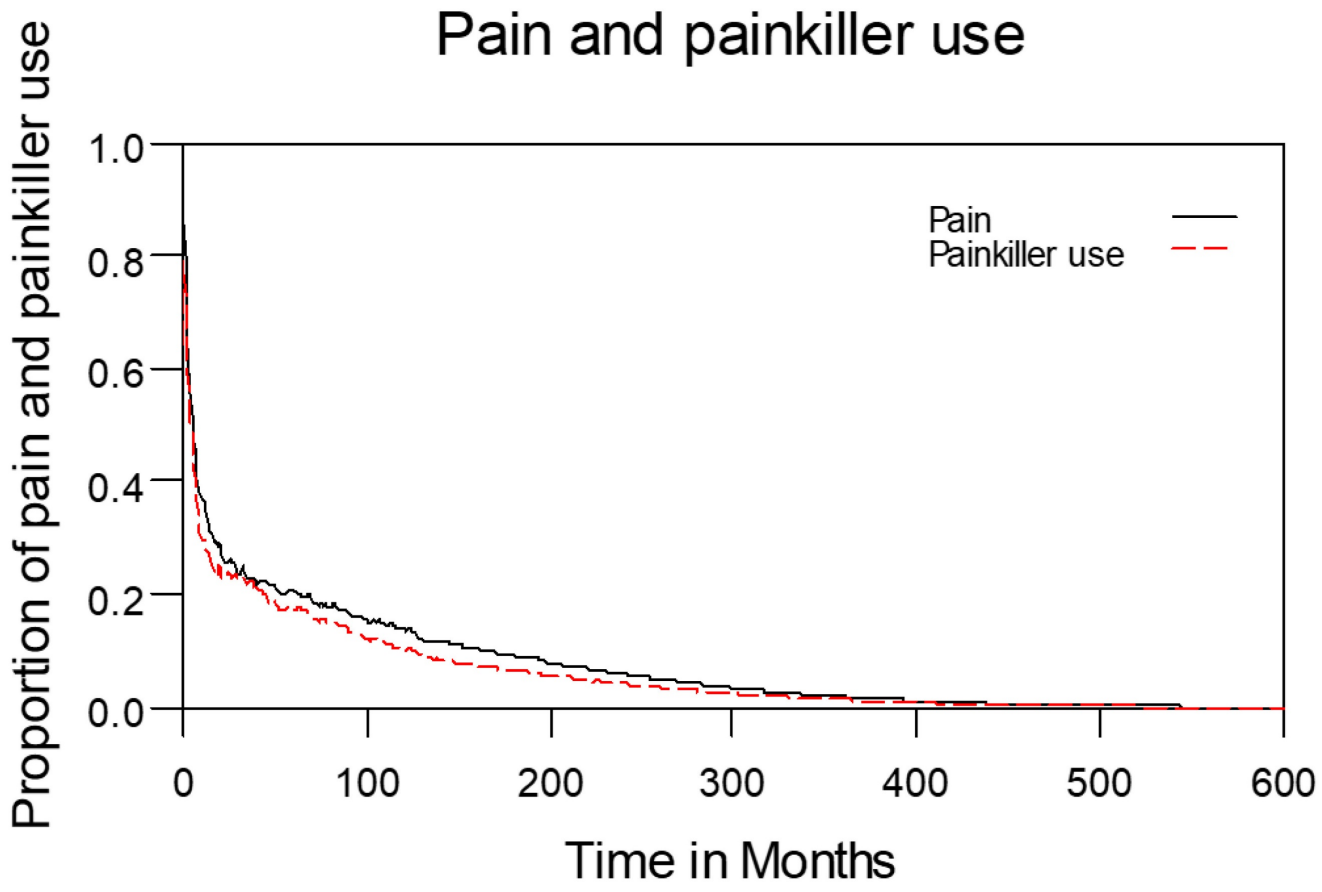
Changes in pain levels and painkiller use.

**Figure 4:**
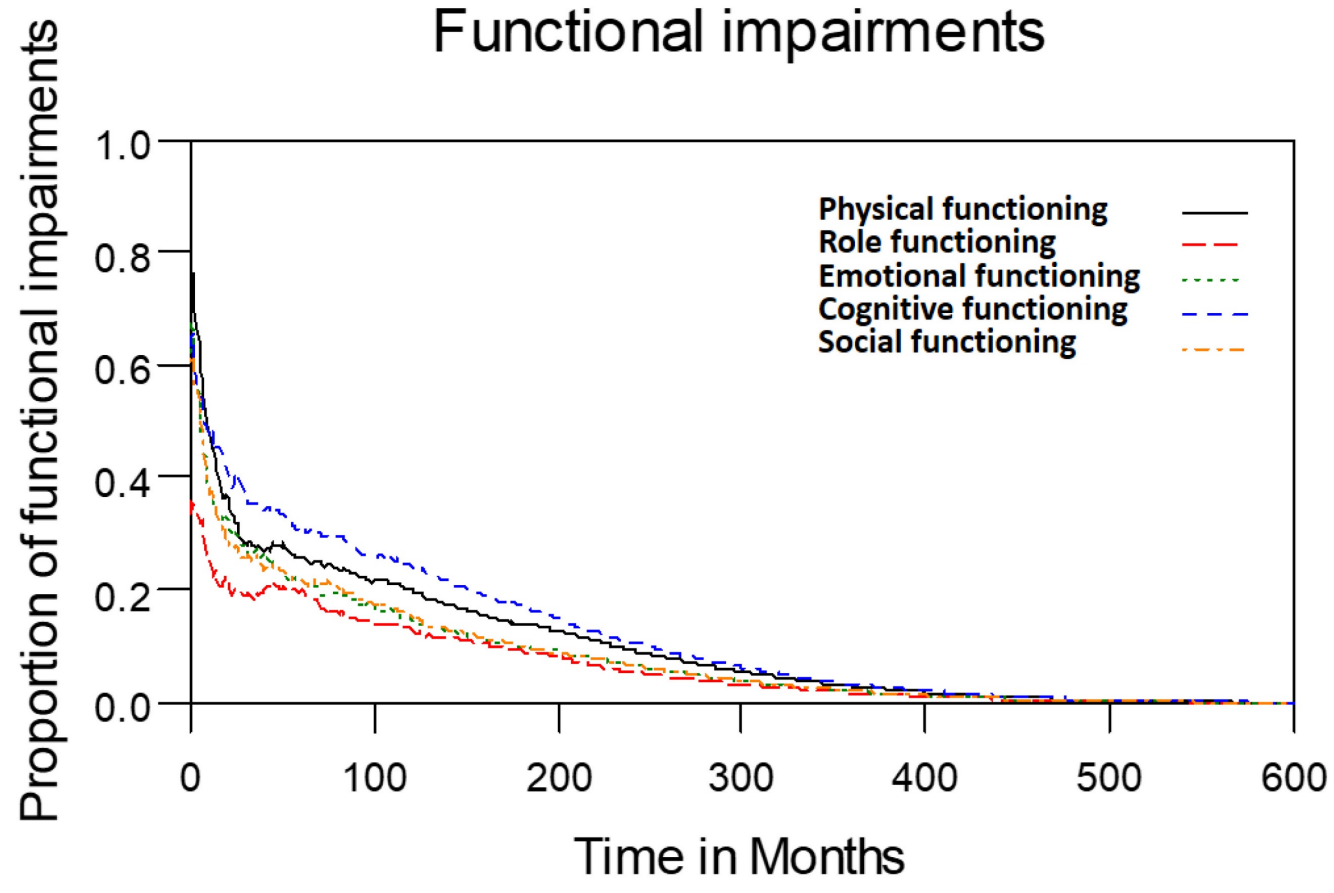
Functional impairments in patients with laryngohypopharyngeal cancer. Duration of functional impairments, as estimated using the area under the quality-adjusted survival curve. Duration of functional impairments (years): physical—4.2; role—2.7; emotional—3.4; cognitive—4.9; social—3.4.

**Figure 5:**
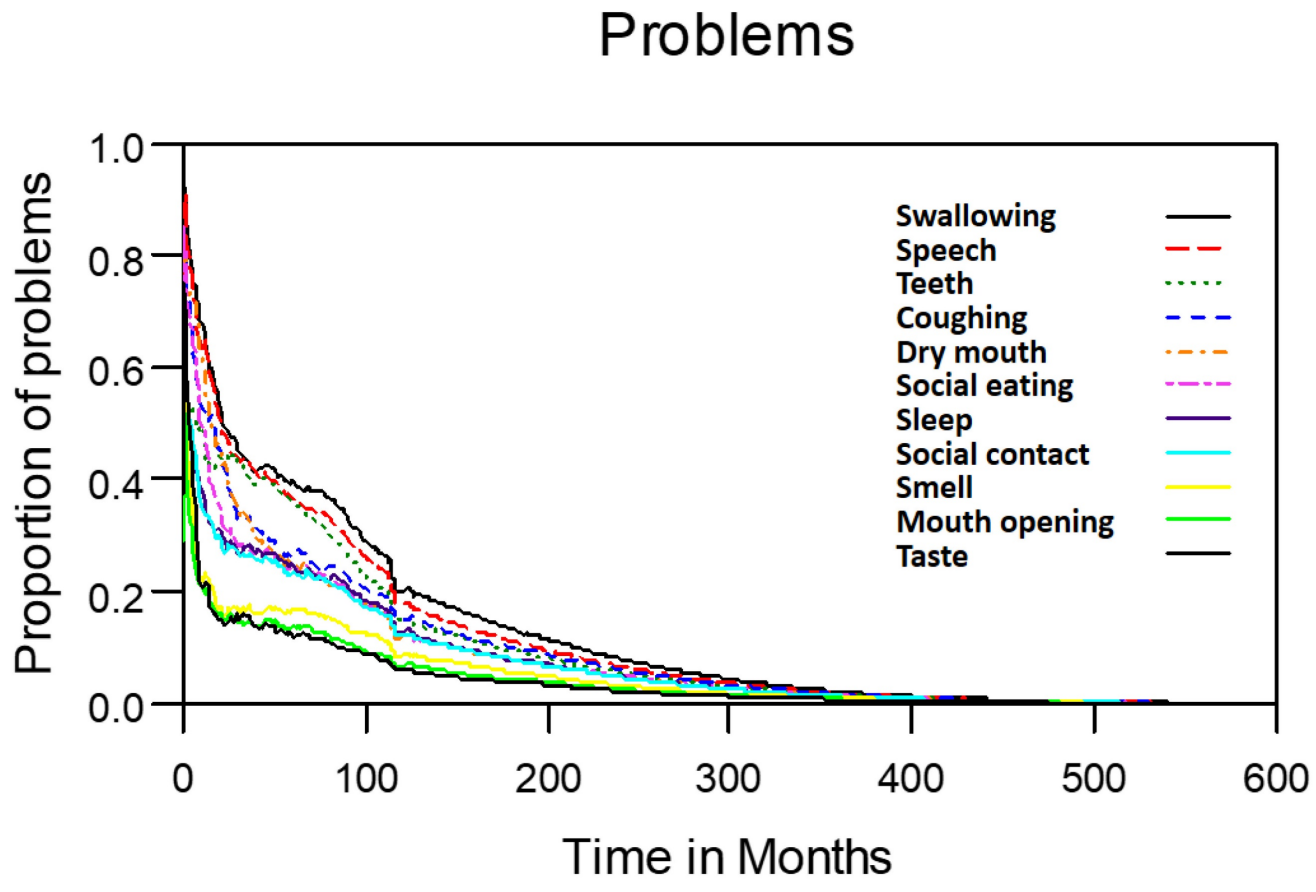
Problems in patients with laryngohypopharyngeal cancer. Duration of impairments or problems estimated using the area under the quality-adjusted survival curve. Duration of functional impairments or problems (years): speech—5.6; swallowing—6.2; teeth—4.8; coughing—4.6; dry mouth—4.2; social eating—4.0; sleep—3.8; social contact—3.6; smell—2.5; mouth opening—2.1; taste—2.0.

**Figure 6:**
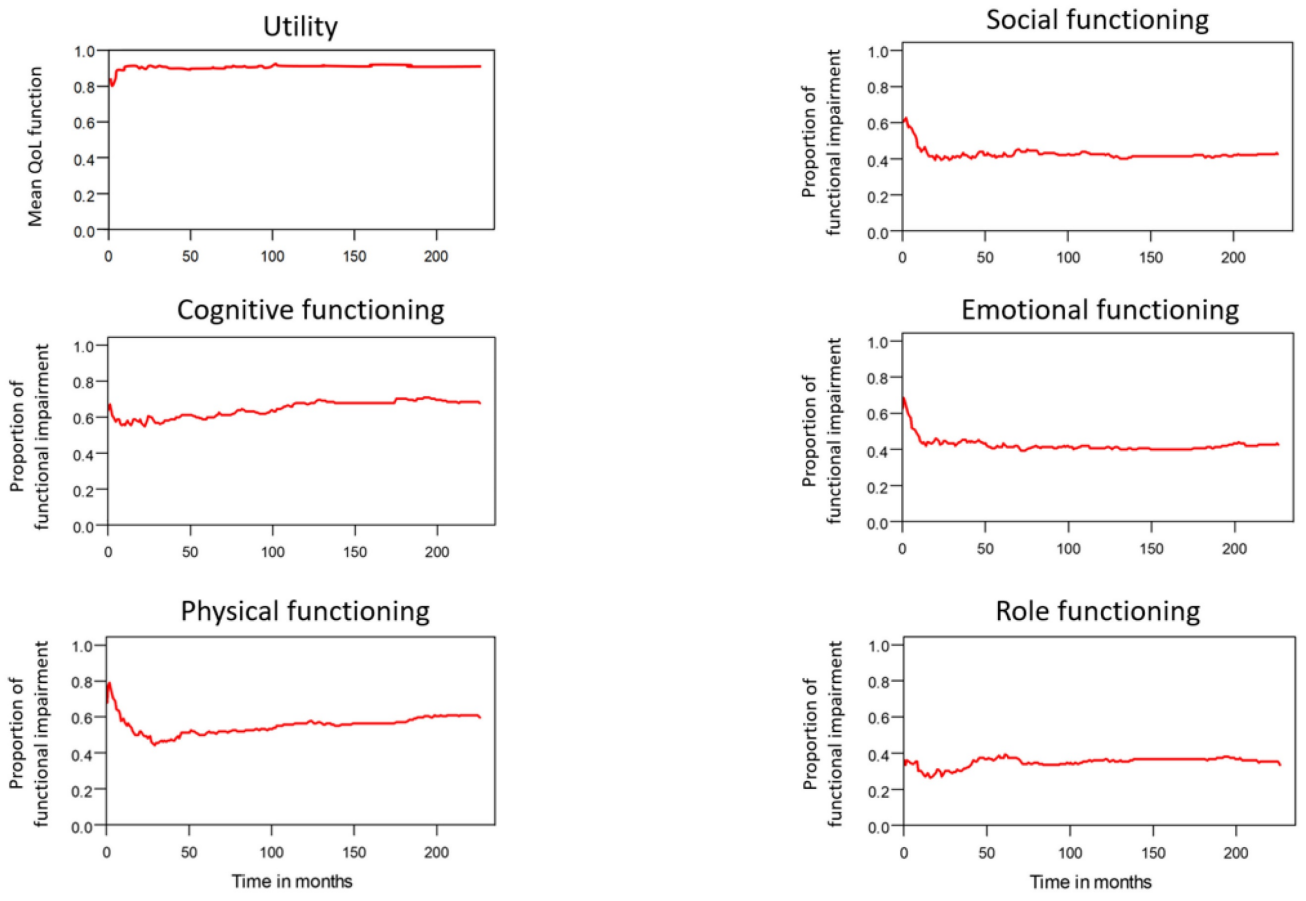
Tends in mean QoL function and functional impairments.

**Figure 7:**
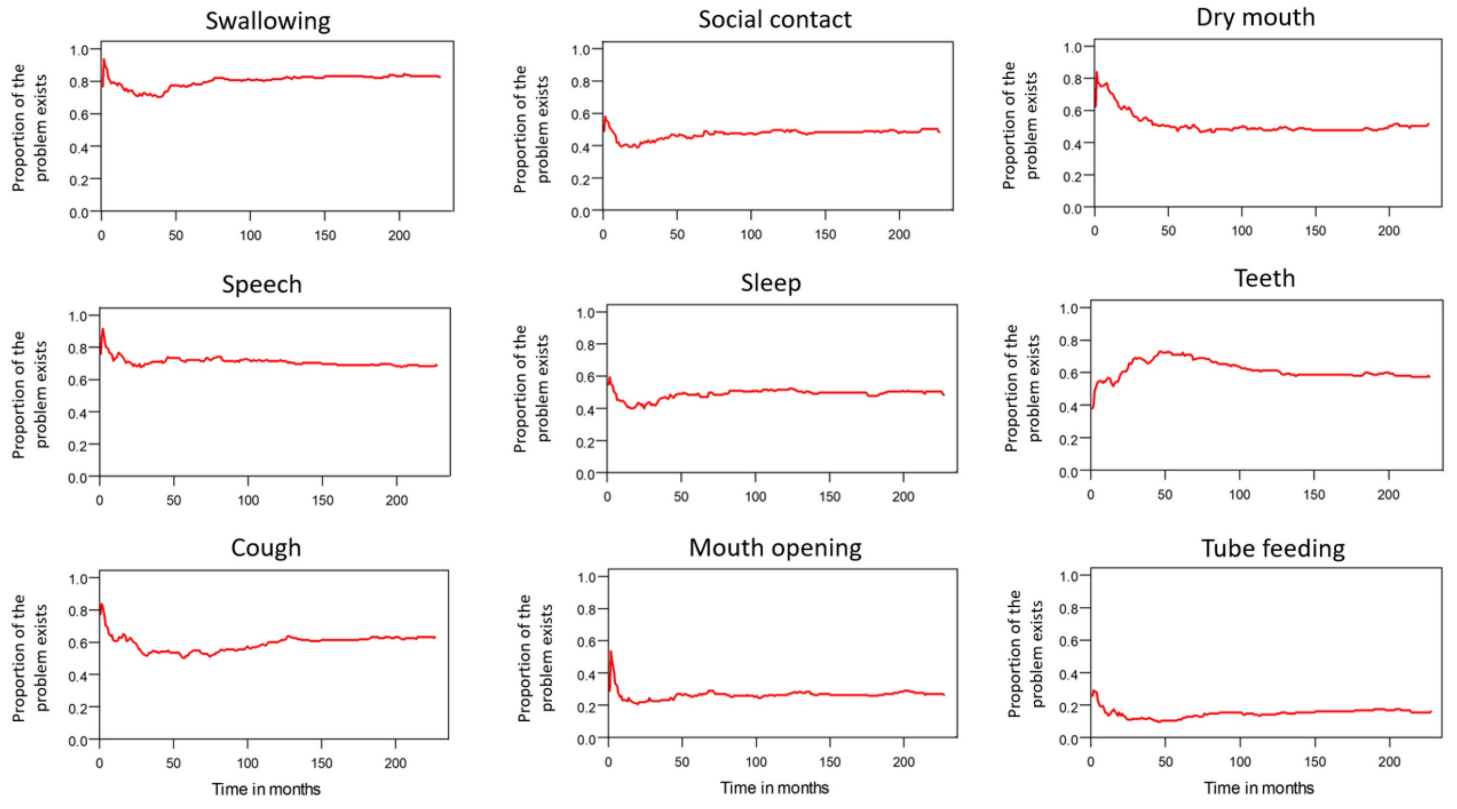
Trends in dynamic changes of various problems.

Table 2 lists the outcomes of the 671 valid questionnaire responses we received from the 232 patients with LHC who completed EQ-5D-3L, EORTC QLQ-C30, and EORTC QLQ-H&N35. The questionnaires were completed a median of 7.0 months after the curative treatments (range: 0 to 225.3 months). Pain scores are related to overall QoL^25^, and our results indicated that the patients with LHC endured pain for an estimated period of 3.2 years (95% CI: 2.4 to 4.9) and relied on painkillers for 2.6 years (95% CI: 2.0 to 3.8; Figure 3). In terms of functional disability, we estimated the durations of impairment in role, physical, cognitive, emotional, and social functioning to be 2.7 (95% CI: 2.1 to 4.0), 4.2 (95% CI: 3.3 to 6.3), 4.9 (95% CI: 3.7 to 7.7), 3.4 (95% CI: 2.7 to 5.1), and 3.4 years (95% CI: 2.6 to 5.2), respectively (Figure 4). The durations of problems related to speech, swallowing, teeth, coughing, dry mouth, social eating, sleep, social contact, smell, mouth opening, and taste were estimated to be 5.6 (95% CI: 4.2 to 7.7), 6.2 (95% CI: 4.4 to 9.3), 4.8 (95% CI: 3.8 to 7.2), 4.6 (95% CI: 3.5 to 7.2), 4.2 (95% CI: 3.4 to 6.0), 4.0 (95% CI: 2.9 to 5.9), 3.8 (95% CI: 2.9 to 5.9), 3.6 (95% CI: 2.7 to 5.5), 2.5 (95% CI: 1.8 to 4.0), 2.1 (95% CI: 1.6 to 3.3), and 2.0 years (95% CI: 1.6 to 2.8), respectively (Figure 5). The projected period of reliance on tube feeding was estimated to be 1.2 years (95% CI: 0.8 to 2.0). In addition, we identified dynamic changes in the prevalence of functional impairments and various problems. The proportions of social, emotional, and physical functional impairments were highest immediately after treatment and gradually decreased (Figure 6). By contrast, the prevalence of cognitive impairment gradually increased before eventually reaching a plateau. Furthermore, the prevalence of most problems decreased over time, with the exception of dental problems, which gradually increased after treatment (Figure 7).

### Validity of Extrapolation

**Figure 8:**
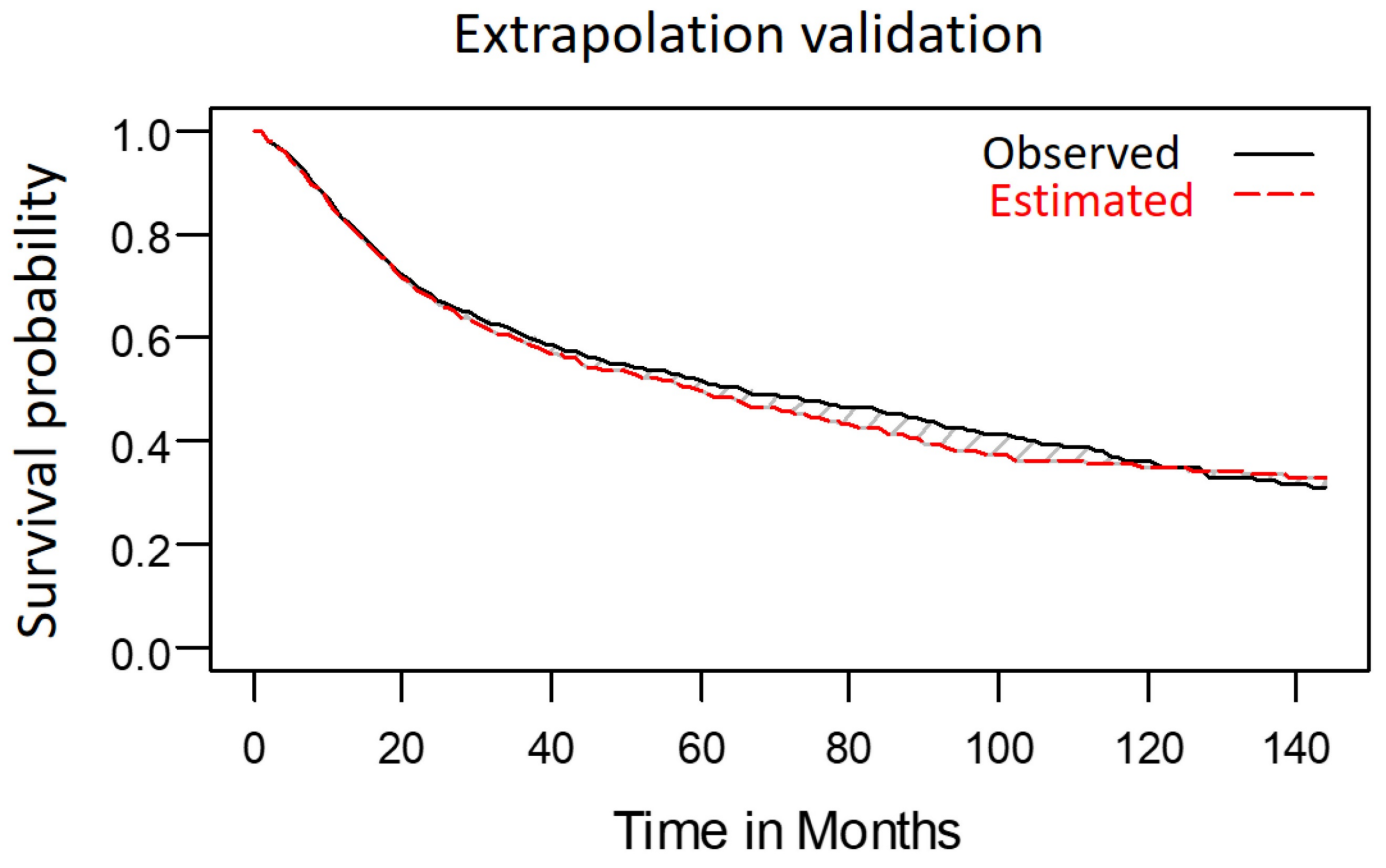
Observed 10-year survival curve aligned well with extrapolated 10-year survival curve.

We compared the extrapolated 10-year OS predictions generated by the model, which was developed using data obtained from 1576 patients over the first 8 years after curative treatment, with the patients' survival outcomes, which were analyzed through the Kaplan-Meier method. The survival outcomes closely matched the estimated survival curve (Figure 8). We determined the mean survival time (±standard deviation) for the patients with LHC to be 67.1 months (±1.6 months). This indicates that the deviation of the estimated survival time from the observed survival time (66.6 ± 1.5 months) after 10 years of follow-up was minimal, at just 0.7%.

## Discussion

A review of the literature indicated that this study is the first to explore lifelong functional disabilities, problems, and QALE in patients who had undergone treatment for LHC. By integrating survival data with QoL information obtained using EORTC QLQ-H&N35, EORTC QLQ-C30, and EQ-5D-3L, we developed comprehensive health profiles that offer an intuitive understanding of the subjective shifts in QoL among patients with LHC. Our findings reveal that for the patients with LHC, the LE was 7.8 years, and the QALE was 7.0 QALYs; the LE and QALE of these patients were respectively 15.7 years and 16.5 QALYs shorter than those of the reference population. Regarding the durations of problems related to LHC, those related to swallowing persisted the longest, lasting for approximately 6.2 years, followed by those related to speech (5.6 years) and teeth (4.8 years). This can be attributed to the enduring and substantial changes in pharyngolaryngeal structures and function that occur after treatment for LHC. By contrast, problems with smell persisted for a fairly short period (2.5 years), and problems related to mouth opening and taste were estimated to last approximately 2.1 and 2.0 years, respectively. Notably, unlike those of most of the investigated problems, the proportion of problems related to teeth increased after treatment, which could be attributed to the long-term effects of RT. Prior studies have documented that cognitive impairment is prevalent in over half of patients receiving treatment for HNC, which aligns with our findings and is likely to have effects on overall QoL and emotional well-being^26^. Our results further indicate that cognitive impairment persisted for approximately 5 years in the study population, highlighting the importance of completing long-term cognitive assessments during posttreatment follow-up. Additionally, our findings reveal that role function impairment persisted for approximately 2.7 years, making it the functional disability with the shortest duration. This shorter duration can be attributed to several factors, including patients actively participating in social or work activities and having strong family support. Of note, our study results revealed that cognitive functioning impairment, physical functioning impairment and swallowing difficulties worsen over time. These findings may be attributed to the natural biological aging process. Additionally, cognitive impairment could be a side effect of RT and chemotherapy^27^-^29^. In the case of locally advanced LHC, the treatment of retropharyngeal lymph nodes often necessitates extending treated volumes towards the skull base, potentially resulting in radiation dose exposure to nearby brain tissues. Some studies indicated inferior neurocognitive outcomes in irradiated HNC patients compared to controls^30^, ^31^. Furthermore, there is evidence suggesting that the risk of neurocognitive dysfunction is a late complication occurring at 1 year or more after RT^32^, ^33^. Cognitive functioning impairment also impacts physical functioning, affecting factors such as walking speed and grip strength^34^. Swallowing problems may result from radiation-induced fibrosis of pharyngeal constrictor muscles^35^, and could also be a consequence of cerebrovascular disease or progressive neurologic disease^36^. To sum up, our study has provided valuable insights into the survival-weighted QoL profiles among patients with LHC, and this information can be instrumental in allocating rehabilitation resources and designing follow-up programs for these patients.

In the context of patients with cancer, QoL profiles can be useful for understanding emotional distress, physical performance, and social functioning^37^. The findings of the present study regarding the SWPSs of patients with LHC may add to the literature on QoL function in such patients in several aspects. First, different cancer stages and treatment modalities can have different effects on certain facets of QoL. For example, the challenges with social interactions and speech were more frequently reported by the patients who underwent surgery, and the patients who received CRT were more likely to experience problems related to sticky saliva, sexuality, and pain^6^. Our study's thorough psychometric assessment of every aspect of QoL provides a comprehensive understanding of the effects of LHC management. Additionally, because notable QoL changes typically occur within the first 2 months after treatment, with most symptom scales stabilizing after a year^38^, this study administered the QoL questionnaires more often during the first 2 months posttreatment to thereby improve the robustness of our estimation of SWPS or QALE. Consequently, our multifaceted assessment of QoL not only captures changes in QoL after LHC treatment but also enhances the usefulness of QoL as an endpoint for evaluating the effectiveness of LHC treatment^39^. Second, patients' subjective views on QoL may change with time^40^. Patients with LHC might experience long-term QoL challenges, such as enduring social functioning impairments and ongoing physical distress, even after achieving disease remission^41^. In this study, by employing an extrapolation method in addition to simulation, we were able to estimate the lifetime QoL of the patients with LHC and thereby elucidate the potential long-term consequences of LHC and LHC treatment. Overall, our study offers support for the notion that estimating SWPSs can be a valuable and comprehensive method for evaluating lifetime QoL in patients with LHC. Accounting for the dynamic nature of QoL and considering its long-term implications can improve the understanding of the challenges faced by patients with LHC and can enable these challenges to be addressed. For instance, patients diagnosed with LHC may find proactive and extended speech and swallowing therapy beneficial, as our study indicates that issues related to speech and swallowing not only have the longest duration but also a high prevalence. For patients with HNC, the implementation of proactive swallowing therapy may contribute to ensuring safe oral intake, preserving adequate nutrition, and ultimately enhancing swallowing function and related QoL^42^, ^43^. Furthermore, incorporating extended physical therapy may contribute to mitigating the observed long-term impairment in physical function^44^. When communicating with patients diagnosed with LHC, the aforementioned information can assist them in choosing treatment modalities with comparable survival outcomes, gaining a clearer understanding of the upcoming challenges and corresponding management, and improving compliance with medical recommendations.

CRT and total laryngectomy were reported to lead to comparably high rates of dysphagia-related QoL changes and morbidity, with these dysphagia-related problems including pneumonia, reliance on oral supplements, and the use of feeding tubes^6^. However, patients who underwent surgery reported significantly more challenges related to their senses of smell and taste, whereas individuals who received CRT experienced problems such as dry mouth and weight loss to a greater extent^6^. Although undergoing extensive surgical procedures often leads to feelings of social isolation and depression^45^, ^46^, our study revealed that problems commonly experienced by surgically treated patients with LHC, such as smell and taste disturbances^6^, ^8^, as well as social and role functional disabilities, had short durations in our study population. Because one-third of our patients underwent primary surgery, the differences between the functional disability problems in our population and those in the literature may partially have been related to differences in treatment modalities. This underscores the importance of formulating treatment-specific psychosocial rehabilitation strategies for patients given a diagnosis of LHC^47^.

We must acknowledge several limitations of this study. First, our SWPS and QALE estimations may have been somewhat exaggerated. Because real QoL often decreases with age^48^, assuming a constant level of QoL during extrapolation may have distorted our results, particularly near the end of the period in which the patients were followed up. Moreover, the patients who survived for a longer time might have completed more questionnaires, potentially leading to higher QoL scores^49^. Second, the loss of QALE might have been overemphasized because we assumed a utility value of 1 for the reference cohort for the entire duration of survival. Additionally, because our study was retrospective, we were unable to obtain detailed clinical and demographic data, including information on underlying comorbidities, marital status, personal drinking and smoking habits, and pretreatment QoL data, all of which could have been valuable for interpreting the results. In addition, according to Chung et al., female patients with a diagnosis of HNC generally have a higher average age at diagnosis, experience a smaller reduction in LE, and have a longer QALE than male patients do^13^. Although we did not control for sex in our study, only four women with LHC completed the QoL questionnaires. This small sample of women emphasizes the need for further investigation into potential gender-related differences in the QoL of patients with LHC. To validate our findings, further large-scale, long-term, prospective studies should be conducted.

## Conclusion

In the patients with LHC in our study, the estimated LE was 7.8 years, and the reduction in LE was 15.7 years. Additionally, the estimated QALE was 7.0 QALYs, and the reduction in QALE was 16.5 QALYs. The findings from the SWPS data indicate that individuals undergoing treatment for LHC face long-lasting functional impairments and persistent problems. To determine whether the insights that can be obtained from QALE and SWPS data can be used to guide the allocation of cancer resources and treatment decision-making for patients with LHC, future large-scale, prospective investigations are warranted.

## Figures and Tables

**Table 1:** Baseline characteristics

Variables	All patients (N=1576)	Patients who completed QOL questionnaires (N=232)
Mean age at diagnosis, years (± SD)	57.4 (± 10.6)	58.9 (±9.6)
Gender		
Male	1525 (96.8%)	228 (98.3%)
Female	51 (3.2%)	4 (1.7%)
Tumor site		
Hypopharynx	1004 (63.7%)	140 (60.3%)
Larynx	572 (36.3%)	92 (39.7%)
T status		
T1	345 (21.9%)	60 (25.9%)
T2	378 (24.0%)	68 (29.3%)
T3	292 (18.5%)	43 (18.5%)
T4	561 (35.6%)	61 (26.3%)
N status		
N0	642 (40.7%)	131 (56.4%)
N1	155 (9.9%)	21 (9.1%)
N2	604 (38.3%)	59 (25.4%)
N3	175 (11.1%)	21 (9.1%)
Cancer stage		
I	265 (16.8%)	53 (22.8%)
II	169 (10.7%)	30 (12.9%)
III	176 (11.2%)	38 (16.4%)
IV	966 (61.3%)	111 (47.9%)
Treatment modality		
Surgical	523 (33.2%)	88 (38.0%)
Surgery + CRT	175 (11.1%)	46 (19.7%)
Surgery + RT	50 (3.2%)	21 (9.1%)
Surgery alone	298 (18.9%)	21(9.1%)
Non-surgical	1053 (66.8%)	144 (62.0%)
CRT	970 (61.5%)	114 (49.1%)
RT	83 (5.3%)	30(12.9%)

*Abbreviations*: SD: standard deviation; QOL: quality of life; RT: radiotherapy; CRT: chemoradiotherapy

**Table 2:** The results of EORTC QLQ-C30 and EORTC QLQ-H&N35

	Mean scores (± SD)
EORTC-QLQ-30	
**Global quality of life scale**	61 (±21)
**Functioning scale**	
Cognitive	81 (±20)
Emotional	84 (±20)
Physical	85 (±20)
Role	84 (±26)
Social	78 (±28)
**Symptom scale**	
Fatigue	26 (±25)
Nausea/vomiting	9 (±17)
Pain	22 (±26)
**Single item**	
Appetite loss	18 (±26)
Constipation	16 (±24)
Diarrhea	7 (±16)
Dyspnea	13 (±21)
Financial impact	24 (±32)
Insomnia	27 (±32)
EORTC QLQ-H&N35	
**Multiple-item symptom scale**	
Pain	16 (±20)
Swallowing ability	30 (±26)
Social contact trouble	16 (±25)
Social eating trouble	23 (±32)
Speech problems	31 (±29)
Less sexuality	40 (±42)
Taste/smell problems	25 (±29)
**Single-item scale**	
Teeth	34 (±37)
Opening mouth	15 (±26)
Dry mouth	36 (±33)
Sticky saliva	37 (±33)
Coughing	32 (±29)
Feeling ill	27 (±32)
Pain killer	50 (±50)
Nutritional supplements	64 (±48)
Feeding tube	20 (±40)
Weight loss	42 (±49)
Weight gain	26 (±44)

Abbreviations: EORTC QLQ, European Organization for Research and Treatment of Cancer Quality of Life Questionnaire; H&N, head and neck; SD, standard deviation.
